# The relationship between myopia and near work, time outdoors and socioeconomic status in children and adolescents

**DOI:** 10.1186/s12889-022-14377-1

**Published:** 2022-11-10

**Authors:** Dariusch Philipp, Mandy Vogel, Manuela Brandt, Franziska G. Rauscher, Andreas Hiemisch, Siegfried Wahl, Wieland Kiess, Tanja Poulain

**Affiliations:** 1 LIFE Child Leipzig Research Center for Civilization Diseases, Leipzig University, Leipzig, Germany; 2 Institute for Medical Informatics, Statistics, and Epidemiology, Leipzig University, Leipzig, Germany; 3 Leipzig University Hospital for Children and Adolescents, Leipzig University, Leipzig, Germany; 4 Institute for Ophthalmic Research, Eberhard Karls University Tuebingen, Tuebingen, Germany; 5 Carl Zeiss Vision International GmbH, Tuebingen, Germany; 6 Center for Pediatric Research, Leipzig University, Leipzig, Germany

**Keywords:** Myopia, Children, Refractive error, Near work, Time outdoors, Socioeconomic status

## Abstract

**Background:**

To investigate environmental and social risk factors for myopia in children and adolescents in Germany.

**Methods:**

1437 children aged between 3 and 18 inclusive were examined as part of the LIFE Child study based in Leipzig, Germany. Information about leisure time activities and social status was ascertained by parents and children in a questionnaire. Refractive status was attained by measuring noncycloplegic autorefraction. Myopia was defined as spherical equivalent (SE) ≤ − 0.75 D. Risk factors were identified using multiple logistic regression analysis.

**Results:**

In multiple logistic regression analysis, myopia was significantly associated with less frequent outdoor activity (“once a week” vs. “twice a week or more”: odds ratio (OR) 4.35, 95% confidence interval (CI) 1.89–9.98, p<0.01) and longer near work sessions (1–2 h vs. < 1 h: OR 1.83, CI 1.10–3.04, p=0.02; > 3 h vs. < 1 h: OR 3.71, CI 1.43–9.61, p<0.01) after adjustment for age, sex and socioeconomic status (SES). Duration of outdoor activity, near work frequency and SES showed no significant association with myopia (p > 0.05). Children with a lower SES were involved in longer periods of outdoor and near work activities but on fewer occasions over the course of the week, although this connection was not significant.

**Conclusion:**

Myopia is associated with environmental factors. The present findings suggest that daily exposure to sunlight and a restriction of long-duration near work activities might protect against pathological eye growth. Prevention strategies should be implemented for children at all ages.

## Background

There remains an underappreciation for the seriousness of myopia amongst the general public. In 2010, uncorrected refractive error (difference between vision impairment at presentation and best-corrected sight) was responsible for one in five cases of blindness and the leading cause of moderate or severe visual impairment [1]. From an economic perspective, according to an estimation by Fricke et al. [2], distance vision impairment caused by uncorrected refractive error, either directly or indirectly, leads to global productivity losses equivalent to USD 202 billion annually. At the same time, the global prevalence of myopia has increased notably in recent decades. A meta-analysis by Holden et al. included 145 studies published since 1995 and found a worldwide increase in the prevalence of myopia from 25.6% in 2000 to 32.3% in 2010 [3]. This trend has been most pronounced in East Asian populations, and particularly in countries with ‘high-pressure educational systems’ such as South Korea, China, Taiwan and Singapore [3], leading to children spending more time indoors out of the sunlight and engaged in near work as part of their studies. A remarkable study from Taiwan estimated an increase in the prevalence of myopia in 12-year-old children from 36.7% in 1983 to 60.7% in 2000, while for young people aged 16–18 in 2000, prevalence had reached 84% [4]. Globally, myopia prevalence is projected to reach approximately 49.8% in 2050 with as many as 5 billion people affected [3].

At birth, children tend to be hyperopic with positive spherical equivalents (SE) [5, 6]. The human eye grows and modifications of the cornea, lens and the axial length of the eye determine refractive status in adulthood. In most cases, these modifications lead to good visual acuity. However, if the process of axial elongation is accelerated, children are more likely to develop myopia.

Why do some eyes grow too fast? The rapid increase in the prevalence [3] and national differences in the progression of myopia [7, 8] suggest that this type of growth cannot be explained by genetic factors. Based on animal experiments finding that intraocular dopamine antagonizes stimuli for eye growth [9] and Jones et al. finding an association of time outdoors with myopia [10], Rose et al. developed the hypothesis that the protective effect of time outdoors is caused by a light-triggered dopamine release in brighter light [11]. Following experiments with vertebrates such as chickens [12] and rhesus monkeys [13] have shown that ambient illuminance has a significant influence on eye growth, although the level of brightness required to have an impact usually only originates in natural sunlight. As such, numerous studies have analyzed the association of sunlight exposure – measured as time spent outdoors – with the prevalence of myopia [10, 11, 14–19]. The suspected association of near work activities with myopia originates from studies from the 19th century, e.g., by Hermann Cohn in 1867. Cohn found that there was a greater prevalence of myopia in children who worked in dark classrooms, and an association between myopia and higher levels of schooling. As students in dark classrooms are required to work at closer distances, and a higher school level demands more studying and reading, he concluded that near work activities were responsible for myopia onset [20]. To date, this hypothesis has been tested in numerous studies examining the correlation of self-reported quantities of near work and myopia [16, 19, 21, 22].

What characteristics tend to be present in children who are at equal risk of becoming myopic? One way to define social class is with the socioeconomic status (SES). SES, or the three associated components education, income and occupational status have been found to be significantly associated with myopia in various studies [15, 23–26].

Myopia prevalence has been reported to be 41.3% among German adults aged 18–35 [5]. However, as this study assessed myopia based on self-reported noncycloplegic measurements, it might overestimate the real value. Although this prevalence remains lower than in some Asian countries, understanding the factors involved in myopia onset is crucial if we hope to appropriately respond to future shifts in prevalence or even reduce the current incidence of myopia. The KiGGS survey, a multicenter study in Germany, explored the relationship of leisure activities with myopia in a large German cohort based on questionnaires in which parents reported whether their children were myopic or not [27]. The research discussed in this paper is intended to supplement this data analysis by determining the relationship between child myopia (identified using autorefraction), time spent outdoors and in near work as well as the role of SES.

## Methods

### LIFE child and study population

The LIFE Child study (clinical trial no. NCT02550236) is part of LIFE, a large-scale research program implemented by the Leipzig University, Germany. LIFE Child is a prospective cohort study carried out at the Leipzig Research Center for Civilization Diseases, which seeks to assess the factors involved in the development of health and non-communicable diseases in young people from the prenatal stage to adulthood. The study launched in July 2011; participants are invited to attend annual follow-ups over a period of 10 years. The population is drawn from the city of Leipzig and its immediate surroundings. Recruitment is conducted in a range of settings, such as schools, kindergartens, local clinics, primary care practices, public health centers and partner studies. The data are collected by a trained multidisciplinary team following standard operating procedures. The majority of participating children have no migratory background (95% of both parents and 92% of all grandparents were born in Germany).

### Eye examination

The data used in this paper were produced in the eye examinations carried out on study participants between the ages 3 and 18 years inclusive. For each subject, three refraction measurements were carried out for both eyes (3 mm pupil diameter and 12 mm vertex distance) using noncycloplegic autorefraction (i.Profiler^®^ plus, Carl Zeiss Vision GmbH, Aalen, Germany [28]). The use of cycloplegics in the measurement of refractive status was not approved by the local Ethics Committee.

For further analysis, each participant was classified for the eye’s refractive error. The refractive error was defined as follows: First the SE was calculated as SE = sphere + cylinder/2; out of the three SE values calculated for each participant, the median value was selected. This practice is suggested by Rauscher et al. who showed that the true refractive error can be approximated by employing the median of repeated measurement in case measurement under cycloplegia is not an option [28]. Myopia was defined as ≤ − 0.75 D.

### Questionnaire data

As part of the basic examination and eye examination, parents and children were asked to answer age-dependent questionnaires. To assess SES, parents reported their highest occupational and educational status and their total household income. Each of these pieces of data was subsequently converted to a score between 1 and 7 inclusive, with the sum of these scores used as a total SES index score for the family. The SES index scores therefore ranged from 3 to 21, with a higher score indicating higher SES. To explore differences in children’s leisure behavior between social classes, we defined three SES groups (low, medium, high) using the SES index as suggested by Lampert et al. in the context of the KiGGS study [29]. Questions about the child’s leisure activities were designed by researchers of the LIFE Child study team. They were answered by parents or, if the child was 10 years old or older, by children themselves. These items produce information about frequency per week and duration per day of reading/writing/drawing tasks (near work) and outdoor playtime. With regard to reading/writing/drawing, the activity with the highest frequency or duration was recorded. Possible responses for the frequency question were “once per week”, “two days a week”, “every two days” and “every day”, while the answer options for duration were “< 1 h”, “1–2 h”, “3–4 h” and “> 4 h”.

### Data analysis

For all data analysis, we utilized the open source application R, version 3.5.3 (R Core Team 2019-03-11). From the 4820 eye examinations conducted to date, each participant’s most recent visit was filtered. This produced a more equal age distribution. Siblings and twins were excluded to prevent genetic bias in our results. After these steps, a total of 1416 examined children remained. A population characteristics table was subdivided into two age groups for comparison purposes as suggested by Schuster et al. [27]. Group differences in the SE of both eyes were tested using *t*-tests.

Using logistic regression models with myopia as the dependent variable, the associations between questionnaire parameters (outdoor play frequency and duration, near work frequency and duration) and myopia were analyzed. These models were then adjusted for age, sex and SES as continuous measure. Finally, we examined whether children from different SES groups showed significantly different leisure time activity patterns with a chi-squared test. The level of significance was set to α = 0.05.

## Results

### Study population

1416 children and adolescents between the ages of 3 and 18 inclusive were included in this study. Table 1 shows the population characteristics.

**Table 1:** Population characteristics of the LIFE Child study sample

Population Characteristics	3–10 years	11–18 years
	*N = 620*	* N = 796*
**Mean age (years):**	7.3	14.9
**Sex:**		
Female	296 (47.7%)	390 (49.0%)
Male	324 (52.3%)	406 (51.0%)
**Myopia:**		
Myopic	24 (3.9%)	171 (21.5%)
Non-myopic	596 (96.1%)	625 (78.5%)
**Socioeconomic status:**		
Low	65 (10.8%)	105 (15.2%)
Medium	272 (45.3%)	366 (52.8%)
High	263 (43.8%)	222 (32.0%)
**Urban environment:**		
No	125 (20.4%)	178 (26.1%)
Yes	488 (79.6%)	505 (73.9%)
**Time outdoors frequency:**		
Every day	461 (79.2%)	264 (45.4%)
Every two days	68 (11.7%)	147 (25.3%)
Two days per week	39 (6.7%)	132 (22.7%)
Once per week	14 (2.4%)	38 (6.5%)
**Time outdoors duration:**		
< 1 h	58 (10.0%)	88 (15.1%)
1–2 h	296 (50.9%)	327 (56.1%)
3–4 h	169 (29.0%)	140 (24.0%)
> 4 h	59 (10.1%)	28 (4.8%)
**Near work frequency:**		
Every day	364 (65.9%)	194 (40.2%)
Every two days	110 (19.9%)	120 (24.9%)
Two days per week	53 (9.6%)	88 (18.3%)
Once per week	25 (4.5%)	80 (16.6%)
**Near work duration:**		
< 1 h	373 (67.6%)	237 (49.6%)
1–2 h	164 (29.7%)	219 (45.8%)
3–4 h	14 (2.5%)	18 (3.7%)
> 4 h	1 (0.2%)	4 (0.8%)

Based on the median SE per eye, the mean SE of all children was − 0.12 ± 0.07 D in the right eye and − 0.11 ± 0.07 D in the left; the median SE values were 0.025 D and 0.018 D, respectively. The *t*-test revealed no significant difference in the refraction values between left and right eyes (p = 0.66). Therefore, the following analysis will refer to refraction data for the right eye only.

Out of 1416 children, 195 (13.8%) children were myopic with a SE ≤ − 0.75 D, and 1221 (86.2%) had a SE > − 0.75 D.

### Myopia and leisure time activities

The results of the univariate logistic regression analysis are displayed in Table 2. It shows significant associations of myopia with low outdoor activity frequency, high near work duration and low near work frequency. Outdoor activity duration was not significantly associated with myopia.

**Table 2:** Univariate logistic regression analysis correlating myopia with leisure time activities and the socioeconomic status

Univariate logistic regression analysis	Odds ratio	95% confidence interval for Odds ratios		p-value
**Socioeconomic status:**	0.93	0.89	0.97	< 0.01
**Time outdoors duration:**				
< 1 h	Reference			
1–2 h	0.90	0.53	1.53	0.70
3–4 h	0.71	0.39	1.29	0.25
> 4 h	0.73	0.32	1.68	0.46
**Time outdoors frequency:**				
Every day	Reference			
Every two days	1.92	1.22	3.05	0.01
Two days per week	2.55	1.60	4.06	< 0.01
Once per week	4.08	2.09	7.95	< 0.01
**Near work duration:**				
< 1 h	Reference			
1–2 h	2.08	1.39	3.12	< 0.01
3–4 h	4.48	1.97	10.21	< 0.01
> 4 h	7.63	1.25	46.77	0.03
**Near work frequency:**				
Every day	Reference			
Every two days	1.37	0.84	2.24	0.20
Two days per week	1.45	0.82	2.56	0.20
Once per week	2.63	1.52	4.56	< 0.01

Table 3 shows the results of the multiple logistic regression analysis. The models from the univariate analysis were complemented by the parameters age, sex and SES of the study participants. Outdoor activity frequency and near work duration maintained a significant association with myopia after this adjustment while near work frequency was no longer significantly associated with myopia. Outdoor activity duration continued to display no significant association with myopia.

**Table 3:** Multiple logistic regression analysis correlating myopia with leisure time activities and SES, adjusted for age, sex and SES

Multiple logistic regression analysis	Odds ratio	95% confidence interval for Odds ratios		p-value
**Socioeconomic status:**	0.95	0.91	1.00	0.06
**Time outdoors duration:**				
< 1 h	Reference			
1–2 h	1.03	0.57	1.87	0.91
3–4 h	1.02	0.52	2.00	0.94
> 4 h	1.25	0.48	3.25	0.65
**Time outdoors frequency:**				
Every day	Reference			
Every two days	1.34	0.81	2.23	0.25
Two days per week	1.33	0.78	2.24	0.29
Once per week	3.05	1.49	6.25	0.01
**Near work duration:**				
< 1 h	Reference			
1–2 h	1.78	1.13	2.80	0.01
3–4 h	3.86	1.56	9.54	0.01
> 4 h	3.15	0.44	22.55	0.25
**Near work frequency:**				
Every day	Reference			
Every two days	0.95	0.55	1.65	0.85
Two days per week	1.06	0.58	1.95	0.84
Once per week	1.18	0.62	2.24	0.61

**Figure 1:**
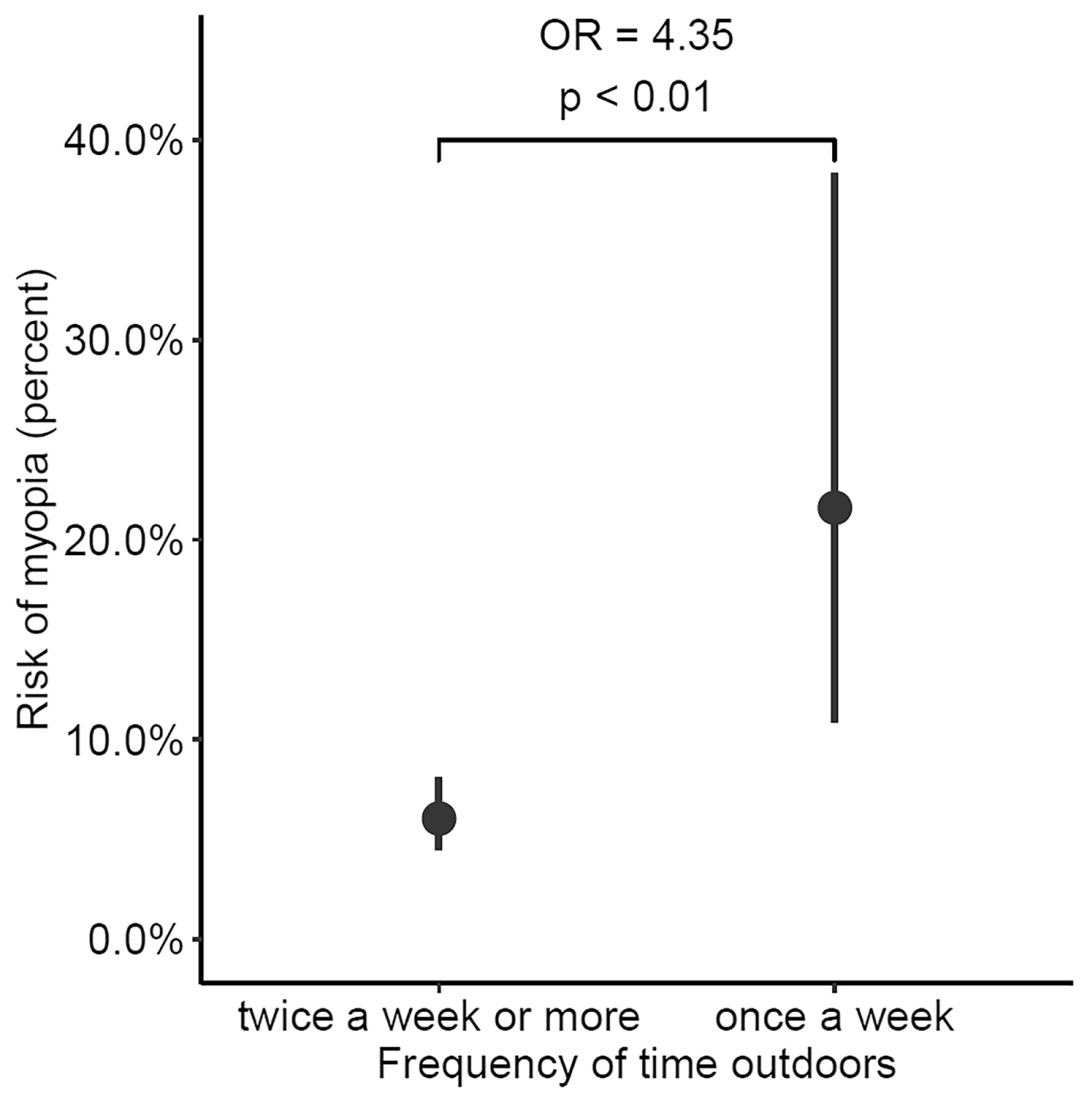
Rate of myopia plotted against reported frequency of outdoor time (days per week)

**Figure 2:**
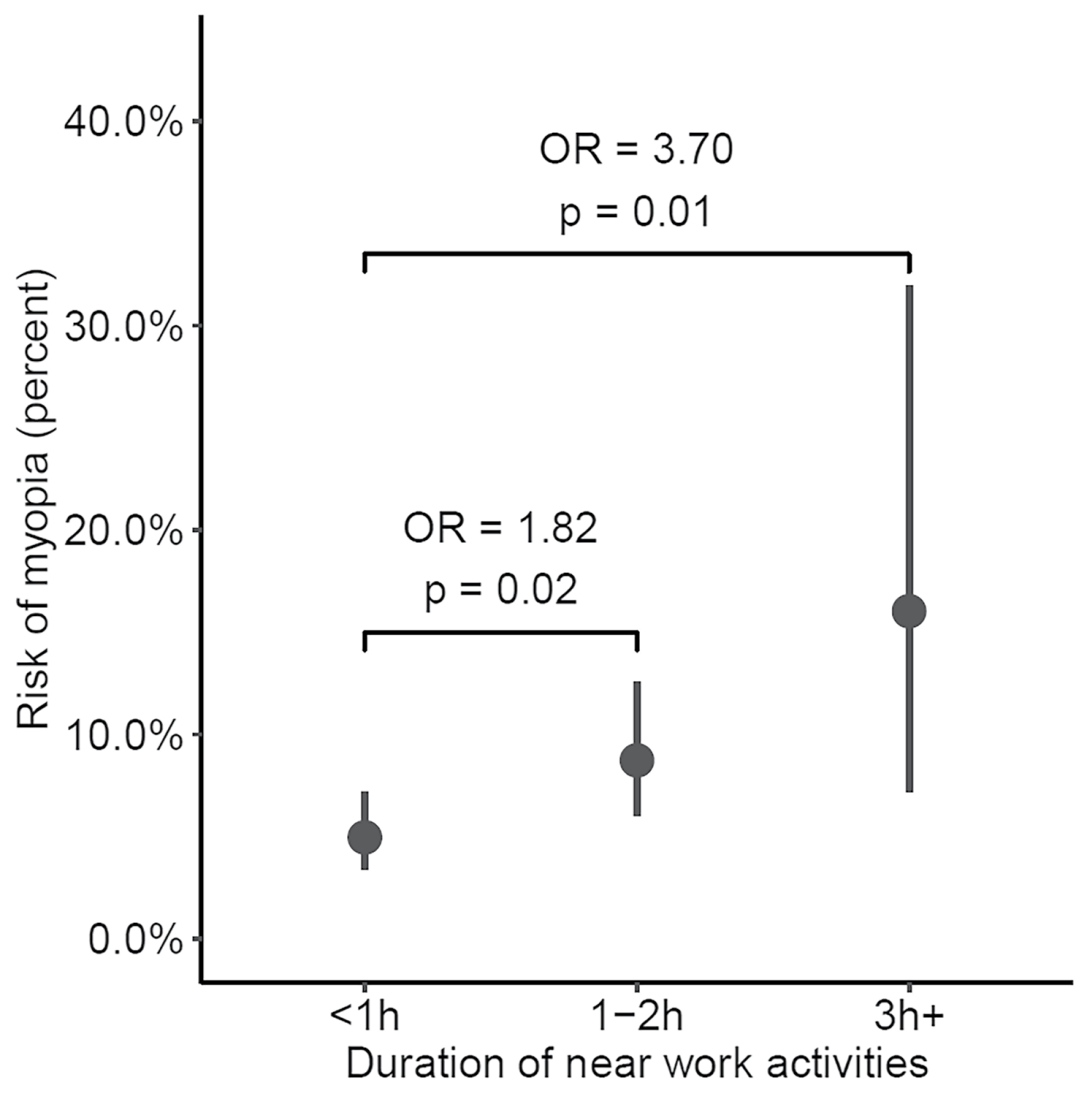
Rate of myopia plotted against reported duration of near work activities on one day

Finally, the two parameters outdoor activity frequency and near work duration were included simultaneously in the same model. Both remained significantly associated with myopia after adjustment. Since outdoor activity frequencies of twice a week or more showed no significant association with myopia (see Table 3), subjects who were reported to spend time outdoors “every day”, “every two days” and “two days a week” were grouped in one group, which was designated “twice a week or more”. This was performed in the same manner with near work durations of “3–4 hours” and “> 4 hours”, merging these response groups into one designated “> 3 h“. The relationships between myopia and outdoor activity frequency and near work duration in this final model are illustrated in Figures 1 and 2, respectively. These show that both low outdoor activity frequency and long near work duration are significantly associated with myopia, even after adjusting for each other as well as for age, sex and SES.

Expressed in terms of probability, children who spend time outdoors once a week were more than four times as likely to be myopic than children who go outside twice a week or more (OR 4.35, 95% CI 1.89–9.98, p < 0.01). Children who engage in near work for longer periods each day were two to four times as likely to be myopic as those who engage in near work for shorter lengths of time (1–2 h vs. < 1 h: OR 1.82, CI 1.10–3.02, p = 0.02; > 3 h vs. < 1 h: OR 3.70, CI 1.43–9.60, p = 0.01).

It was then checked whether the associations between myopia and the various leisure time activity parameters varied with the age of the participant, but interactions of age with outdoor activity frequency and near work duration all failed to reach significance (all p > 0.22). This was the case both when the subjects were divided into age groups – preschool (3–5 years), elementary school (6–10 years) and high school (11–18 years) – and when the interactions with age were tested as a continuous variable. Interactions of sex (all p > 0.20) and SES (all p > 0.08) with outdoor activity frequency and near work duration were not significant either.

### Myopia and socioeconomic status

**Figure 3:**
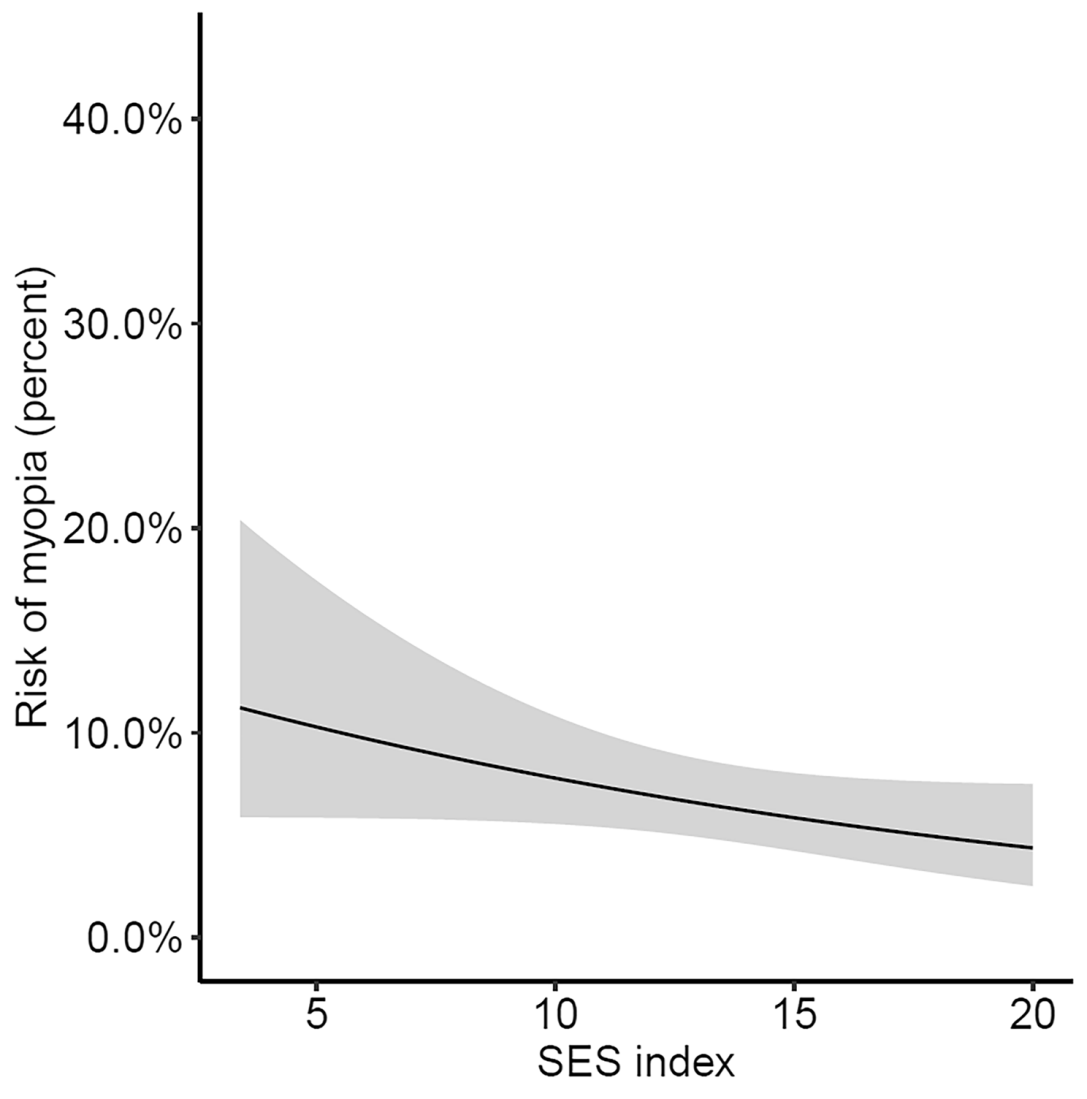
Rate of myopia as a function of the SES index

The distribution of children between SES groups is displayed in Table 1. In univariate logistic regression analysis, higher SES was significantly associated with a lower likelihood of myopia (see Table 2). However, after correcting for age and sex (see Table 3), this association was no longer significant. Figure 3 illustrates the relationship between the SES index and myopia in the afore mentioned final model with adjustment for age, sex, outdoor time frequency and near work duration. The rate of myopia decreased with higher SES values, although this association did not reach significance (OR 0.94, CI 0.88–1.01, per + 1 at the SES index scale, which corresponds to an OR of 0.73 per + 5 on the SES index scale, p = 0.08).

Children with a low SES played outside on fewer days per week (every day: 59.8% with low SES vs. 67.7% with high SES; once a week: 7.2% vs. 4.3%; p = 0.04, chi-squared test), but for longer time periods (1–2 h: 44.3% vs. 59.6%; > 4 h: 12.4% vs. 5.0%; p = 0.04) than children with a high SES. Furthermore, children with a low SES were engaged in near work activities on fewer days per week (every day: 42.9% vs. 60.3%; once a week: 14.3% vs. 7.8%; p = 0.09), but for greater amounts of time (< 1 h: 54.9% vs. 63.3%; > 4 h: 1.2% vs. 0.4%; p = 0.57) than children with a high SES. Although these relationships are comprehensible, the differences between the SES groups were not significant (p > 0.05) for the parameters “near work frequency” and “near work duration”.

## Discussion

The LIFE Child study is the first study in Germany to work with refraction data in a cohort of children and adolescents aged 3–18 years. A detailed analysis of the refractive status of children in the LIFE Child cohort is described elsewhere [30]. The prevalence of myopia in this cohort (3.84% in the 3- to 10-year-old age group and 21.5% in 11- to 18-year-olds) is similar to the findings of Schuster et al. in another German cohort, which estimated that 5.4% of 3- to 10-year-olds and 21.1% of 11- to 17-year-old children were myopic [27]. Compared to Schuster et al., children with a high SES are overrepresented in our population (our population vs. Schuster et al.: 43.8% vs. 27.1% in the younger cohort and 32.1% vs. 24.7% in the older cohort), and children with a low SES are underrepresented (10.6% vs. 27.0% in the younger cohort and 15.3% vs. 26.2% in the older cohort) [27]. However, we were still able to include more than 120 children with a low SES for robust data analysis.

Myopia prevalence was higher in older than in younger children. Less frequent time outdoors, shorter time outdoors durations and longer near work activities in older children, probably due to a growing amount of school hours and homework as well as a change in the way older children spend their remaining leisure time, might be possible reasons for this effect. While young children like to play outside, adolescents tend to be more involved in (social) media. The findings that outdoor time becomes shorter and less frequent in older children is in line with several other studies, e.g. Gao et al. [31], Shah et al. [32] and Enthoven et al. [19]. A higher near work frequency of younger children is rather puzzling as we defined near work as reading, writing and drawing and children only learn to read at the age of six years. A social desirability bias will presumably have a substantial influence as questionnaire data was ascertained by parents for children younger than 10 years.

With regard to time spent outdoors or engaged in near work, we distinguished between the length per day and the frequency per week of leisure time activity, which enabled us to investigate more precisely which aspect of leisure time behavior is more important with respect to myopia.

### Time outdoors

Since Jones et al. found that an increased amount of time spent outdoors was negatively associated with myopia [10], many other studies followed, mostly detecting the same relationship of myopia and time outdoors, regardless of whether a cross-sectional [11] or longitudinal [14] study design was chosen. The results are consistent for different populations, e.g., Dutch [19], Spanish [17] or Chinese [15, 16]. A global meta-analysis by Xiong et al. in 2017 included 25 studies with 34420 subjects and found negative associations between the amount of time spent outdoors and the incidence and prevalence of myopia [18].

In multivariate analyses, our data suggest that the frequency of daylight exposure is more important than the duration concerning myopia onset. The total time spent outdoors throughout a day was not significantly associated with myopia. Based on our data, we cannot tell whether time outdoors was spent in sunlight or not. Especially in winter, children might spend time outdoors without being exposed to sunlight. At the same time, children who played outside multiple times per week were less likely to be myopic than children who only played outside once a week. This finding indicates that, when achieved on a daily basis, even a short stimulus of sunlight might inhibit eye growth and go along with a reduced myopic shift. Based on animal experiments [33, 34], Wallman and Winawer reported in a review that short, but intermittent eye-growth-inhibiting signals in between lasting periods of growth-inducing signals have a disproportionately high protective effect with regard to myopic shift [35]. A frequent daylight exposure operates as this intermittent growth-inhibiting signal. An experiment on chicks by Lan et al. supports this hypothesis, finding that low frequency cycles of bright light provide a stronger inhibiting effect against deprivation myopia than exposures to bright light for 5 h or more [36]. The dominant association of outdoor frequency compared to outdoor duration with myopia in our study does not contradict but rather complement current literature as it offers a more distinguished insight into the underlying mechanisms responsible for the significant association of time outdoors with myopia in the studies mentioned above [10, 11, 14–19].

Based on evidence from observational studies like these, He et al. explored the effect of prevention measures in 6-year-old Chinese primary school children in a cluster-randomized intervention-controlled trial. The intervention group (952 children) was assigned one additional 40-minute class each school day and parents were encouraged to engage their children in more outdoor activities after school. The control group (951 children) followed their usual activity pattern. After three years, the intervention group showed a significantly lower cumulative incidence rate of myopia (30,4%) and change in the SE refraction (− 1.42 D) than the control group (39.7% and − 1.59 D) [37], leading to the conclusion that onset and progression of myopia can be controlled through such interventions. Comparable intervention studies from Taiwan also found a protective effect of increased outdoor activity time against myopia onset [38, 39].

### Near work

An inconsistent definition of near work among authors complicates the process of comparing results and drawing a final conclusion on the association between near work and myopia. Even if we exclude studies that include mid-distance activities as part of their near work investigated, such as watching TV or computer time, and those that examined preschool children, there is still no clear picture. A longitudinal study from the United Kingdom [40] and a cross-sectional study from the Netherlands [19] both defined near work as “reading” and found a positive association between near work and myopia. A cross-sectional study from Beijing, on the other hand, could not find an association between near work (activities < 50 cm distance) and myopia [16]. A study by Ip et al. is notable for the examination of intensive near work: Longer time periods (> 30 min) spent on reading for pleasure and a close reading distance (< 30 cm) were significantly associated with a more myopic refraction [21].

We defined near work as “reading, drawing or writing”. In multivariate analyses, a greater amount of near work per day was positively associated with myopia, but a greater frequency of near work activity per week was not. As suggested by Ip et al., the intensity of near work with constant accommodation seems to have a larger effect on myopia genesis than the frequency with which such near work takes place. This is concordant with the assertion by Wallman and Winawer, as mentioned above [35]. The interruption of near work activities is a signal that slows down eye growth. In our data, this is demonstrated by significant differences in the effect, in terms of myopic shift, of longer near work periods compared to shorter periods. Huang et al. from Taiwan reported similar results, finding that taking breaks to interrupt near work activities lasting 30 min or more led to significantly less myopia after 6 months than was the case with children who did not interrupt long sessions (> 30 min) of near work [22]. Intervention-controlled studies with school classes of varying durations could be used to confirm whether there is a causal effect. As such, these insights may result in the development of measures to prevent myopia in children.

### Socioeconomic status

In the present study, the SES measure is composed of three variables: household income, parental education and parental occupation. Irrespective of what combinations of these variables have been used in previous studies, a high SES has usually been associated with a higher prevalence of myopia. In a study with 1st- and 4th-grade children from Beijing, those with a higher SES had a significantly higher risk of being myopic [15]. Saw et al. share similar results in 7- to 9-year-old children from Singapore [23]. One of the only exceptions to this association was found in 5711 6-year-old children from Rotterdam. Tideman et al. found that a low family income and a low educational level on the part of the mother was significantly associated with a higher prevalence of myopia in children [24]. In all three mentioned studies, the associations did not remain significant after adjusting for outdoor activity and near work time [15, 23, 24], indicating that the effect of SES might be explained by differences in near work behavior and outdoor activity in different SES groups. Many other studies, which were unable to adjust for near work and outdoor time, retained a significant association in multivariate analyses [25, 26].

Our analyses indicate the same relationship between SES and myopia as did the findings of Tideman et al.: the lower the parents’ SES index score, the greater the risk that their children will be myopic. As in previous studies [15, 23, 24], the association was no longer significant after adjusting for the frequency of outdoor activity and the duration of near work.

In our cohort, children with a higher SES tended to play outside more often and to spend shorter periods of time engaged in near work than children with a lower SES. These relationships are indicative of healthier leisure time habits, with regard to eye health, among children from a higher social class. A greater awareness of lifestyle risks among families with a higher SES may be responsible for this. Tideman et al. reported – in reference to the Rotterdam-based cohort – that children with a low SES spend more time engaged in indoor activities with less compensation provided by outdoor exposure and that lifestyle factors explained 71% of the increased risk for myopia in children with a lower SES [24]. Most other studies indicate that children with a higher SES spend less time playing outside and more time in near work activities, explaining the comparatively inverse association of myopia with SES.

### Limitations

The major limitation of this study is that we were unable to use cycloplegics during eye examination. To reduce an overestimation of the prevalence of myopia, we set the cutoff for myopia as − 0.75 D and used the median value out of three measurements per eye. Although myopia prevalence is comparable to the German KiGGS study cohort [27], it is unclear whether these measures exclude some genuine myopic children and whether they effectively eliminate the number of pseudo-myopes in our cohort, so the true cycloplegic value remains unknown.

Another parameter reflecting near work activity is smartphone use, which was included as a separate question in our assessment. However, to date, we do not have a sufficient amount of data to investigate smartphone use, and regrettably, this shift in near work behavior in children in the past 10–15 years is not represented in the present results.

A further limitation is the large age range of the included children. Even if all associations were adjusted by child age, confounding cannot be completely excluded.

Although recruitment of study participants was conducted with diverse methods, a recruitment bias remains an issue. Citizens who do not speak German as well as families with a low SES are underrepresented in our study cohort.

The questionnaires used to assess near work and time outdoors were designed by our researchers and were not validated. Furthermore, depending on the age of the participant, questionnaires were completed either by the parent or, for young people aged 10 and upwards, by children themselves. Parents and the young participants may have different perspectives and estimate leisure time activity differently. In addition to questionnaire bias in general, we should consider the potential for a recall bias as well as a social desirability bias [41], especially concerning questions about outdoor time.

## Conclusion

Given the rapid increase in myopia prevalence in East Asian countries, it is likely that environmental factors contribute to the genesis of myopia to a large degree. Our findings suggest that a daily exposure to sunlight and restricting the duration of uninterrupted near work might protect against pathological eye growth. Furthermore, in our cohort, children from a higher social class tended to be less myopic. Considering the vast economic burden caused by myopia, and the apparent cost-effectiveness of intervention, prevention strategies should be established for children at all ages.

## Data Availability

The datasets generated and/or analyzed during the current study are not publicly available due to ethical restrictions. The LIFE Child study is a study collecting potentially sensitive information. Publishing data sets is not covered by the informed consent provided by the study participants. Furthermore, the data protection concept of LIFE requests that all (external as well as internal) researchers interested in accessing data sign a project agreement. Researchers that are interested in accessing and analyzing data collected in the LIFE Child study may contact the data use and access committee (forschungsdaten@medizin.uni-leipzig.de).

## Abbreviations

SE: spherical equivalent
SES: socioeconomic status
OR: odds ratio
CI: confidence interval
